# Editorial to volume 71 issue 1

**DOI:** 10.1007/s42360-018-0023-1

**Published:** 2018-05-09

**Authors:** M. Anandaraj

**Affiliations:** Indian Phytopathology, Indian Phytopathological Society, New Delhi, India

Dear Readers

The Indian Phytopathology Journal enters the next volume, a welcome change has occurred and the Indian Phytopathological society is joining hands with Springer in publishing this journal. No doubt it would bring better visibility than when it was first brought on line. The quality of articles depends up on the researchers and I urge each one of you to send the best papers to Indian Phytopathology.

In this issue there are twenty-one articles including two review articles one invited and another contributed. In the invited review Indu sawant has dealt with microorganisms for sustainable disease management drawing examples liberally from her own experience. In the next review Manjunatha et al. have extensively covered *Ascochyta* blight of chick pea starting from pathogen diversity, host resistance and molecular breeding for resistance. There are fourteen research articles and five short communications. The crops include the cereals wheat and rice; vegetable crops include potato, tomato, cauliflower and one each from oil crop sesame, one fibre crop jute and a medicinal crop. The major pathogens in these articles are; *Alternaria, Bipolaris, Ceratocystis, Fomes, Fusarium, Macrophomina, Podosphaera, Pythium* and *Ustilaginoidea.* The articles emphasize on pathogen diversity, screening and locating new sources of resistance; Epidemiology and management strategies besides secondary metabolites. There is a fair share of beneficial microorganisms including Arbuscular Mycorrizal fungi with plant growth promoting rhizobacteria and the versatile organism with multifarious activities *Trichoderma.* The results indicate a sustainable non-chemical means of disease management as emphasized in the invited review article as well.

Some of the articles looked like repetitive research on soil borne pathogens like *Pythium* causing soft rot albeit with new chemicals.

In the climate change scenario, new pathogens and their races are likely to have significant effect and that is addressed in the article on *Puccinia triticina.*

My sincere thanks are due to editorial team and business manager who have made all efforts to collaborate with Springer and maintain the periodicity of Indian Phytopathology. I would also like to thank all the reviewers for their help in maintaining the quality of publications.

I would like to urge all the researchers to send their latest and best results to Indian Phytopathology to enhance the quality of the Journal.

